# Activation of NQO1 in NQO1*2 polymorphic human leukemic HL-60 cells by diet-derived sulforaphane

**DOI:** 10.1186/s40164-016-0056-z

**Published:** 2016-09-13

**Authors:** Joseph M. Wu, Ardalan Oraee, Barbara B. Doonan, John T. Pinto, Tze-chen Hsieh

**Affiliations:** Room 147, Department of Biochemistry and Molecular Biology, Basic Sciences Building, New York Medical College, 15 Dana Road, Valhalla, NY 10595 USA

**Keywords:** Sulforaphane, NQO1, HL-60, Leukemia

## Abstract

**Background:**

The NAD(P)H: quinone oxidoreductase (NQO1) confers protection against semiquinones and also elicits oxidative stress. The C609T polymorphism of the *NQO1* gene, designated NQO1*2, significantly reduces its enzymatic activity due to rapid degradation of protein. Since down regulation of NQO1 mRNA expression correlates with increased susceptibility for developing different types of cancers, we investigated the link between leukemia and the NQO1*2 genotype by mining a web-based microarray dataset, ONCOMINE. Phytochemicals prevent DNA damage through activation of phase II detoxification enzymes including NQO1. Whether NQO1 expression/activity in leukemia cells that carry the labile NQO1*2 genotype can be induced by broccoli-derived phytochemical sulforaphane (SFN) is currently unknown.

**Methods and Results:**

The ONCOMINE query showed that: (1) acute lymphoblastic leukemia and chronic myelogenous leukemia are associated with reduced NQO1 levels, and (2) under-expressed NQO1 was found in human HL-60 leukemia cell line containing the heterozygous NQO1*2 polymorphism. We examined induction of NQO1 activity/expression by SFN in HL-60 cells. A dose-dependent increase in NQO1 level/activity is accompanied by upregulation of the transcription factor, Nrf2, following 1–10 μM SFN treatment. Treatment with 25 µM SFN drastically reduced NQO1 levels, inhibited cell proliferation, caused sub-G_1_ cell arrest, and induced apoptosis, and a decrease in the levels of the transcription factor, nuclear factor-κB (NFκB).

**Conclusions:**

Up to 10 μM of SFN increases NQO1 expression and suppresses HL-60 cell proliferation whereas ≥ 25 μM of SFN induces apoptosis in HL-60 cells. Further, SFN treatment restores NQO1 activity/levels in HL-60 cells expressing the NQO1*2 genotype.

## Background

Cancer is the second most frequent cause of death worldwide, with a projected 70 % rise over the next two decades reaching an estimated 24 million new cancer cases per year by 2035 [1, 2]. Genomic instability caused by dysregulation of intracellular redox status that gives rise to sustained oxidative stress is considered to be a risk factor for cancer [3, 4]. Quinones, present in the intra- and extracellular compartments of almost all tissue types, contribute to cellular oxidative stress if they participate in one-electron transfer reactions with concomitant formation of highly reactive semiquinones [5]. Accordingly, half-reduced quinones can participate in the carcinogenic process by causing depurination of DNA nucleotide bases [6–8].

NAD(P)H:quinone oxidoreductase (NQO1) catalyzes two-electron reduction of quinones [9] to products that are readily excreted by conjugation with sulfate or glucuronic acid [10, 11]. Thus, NQO1 provides protection against oxidative stress and exhibits versatility as a cytoprotective enzyme [12, 13]. In addition to conferring cellular protection, NQO1 counteracts DNA damage by preventing semiquinone generation caused by exposure to certain xenobiotics and carcinogens, in particular, benzo[*a*]pyrene-derived dihydrodiol epoxides and *o*-quinones derived from oxidized catechols [14–17]. NQO1 also controls cell proliferation and induction of apoptosis by stabilizing the tumor suppressor protein, p53 [18, 19].

Multiple single-nucleotide polymorphisms (SNPs) have been reported to cause a dysfunctional *NQO1* gene [20, 21]. One extensively studied and significant polymorphic variant is NQO1*2, which is characterized by a C609T (rs1800566, Pro187Ser) polymorphism of the *NQO1* gene [12, 22, 23]. Individuals carrying heterozygous or homozygous alleles of this genetic variant manifest with the highly unstable NQO1*2 isoform, that undergoes enhanced polyubiquitination and proteasomal degradation, resulting in decreased or complete loss of NQO1 activity and a consequential increased susceptibility to cancer [24, 25]. The NQO1*2 polymorphism has been widely investigated but limited information is available on whether the NQO1*2 genotype is associated with specific types of cancers and whether it can be a useful prognostic indicator for efficacy of chemoprevention in specific cancer models. In this communication, we examined microarray data from the Oncomine database to investigate the correlation between under-expression of NQO1 and human leukemia. An apparent association of reduced NQO1 expression was found among different types of leukemia specimens compared to normal. Previous studies show that extracts of cruciferous vegetables, such as broccoli, contain antioxidant phytochemicals that induce Nrf2-regulated cytoprotective genes including NQO1 and other phase II detoxification enzymes [26–28]. However, the ability of the isothiocyanate SFN to induce NQO1 functionality in NQO1*2 cancer cells remains largely unknown. Accordingly, we perform studies to address the following questions: (1) can the expression level of NQO1 provide a surrogate measure for certain cancers and (2) can broccoli-derived SFN activate NQO1 expression in NQO1*2 cells? We provide evidence that SFN [29–31] can induce NQO1 enzymatic activity and expression of the NQO1*2 polymorphic variant. SFN also upregulates a number of cytoprotective enzymes involved in maintaining intracellular redox capacity [32, 33].

## Methods

### Oncomine analyses

The freely available web-based microarray dataset platform, Oncomine [34, 35], was used to examine the fold change of NQO1 mRNA expression levels among leukemia samples. The keywords ‘NQO1’, ‘leukemia’, ‘HL-60’ and ‘clinical specimen’ were used to obtain the initial data for evaluation. A further quality check was conducted using p < 0.01 as the statistical threshold; only samples with NQO1 expression data that met this level of significance were included in the analyses.

### Reagents

Sulforaphane was purchased from LKT Laboratories (St. Paul, MO). Bovine serum albumin (BSA), menadione, dicoumarol and NADPH were purchased from Sigma-Aldrich Corporation (St. Louis, MO). Primary antibodies against NQO1, Keap1, Nrf2, NFκB p50, NFκB p65, IκB, caspase 2, caspase 3, bcl-2, bax, actin, histone H1 and secondary antibodies were purchased from Santa Cruz Biotechnology, Inc (Santa Cruz, CA). Anti-poly (ADP-ribose) polymerase (PARP) antibody was purchased from Biomol International, L.P. (Plymouth Meeting, PA). Fetal calf serum, RPMI-1640, penicillin and streptomycin were purchased from Cellgro, Inc (Herndon, VA). All other chemicals and solvents used were of analytical grade.

### Cell culture and growth inhibition assay

**Fig. 1:**
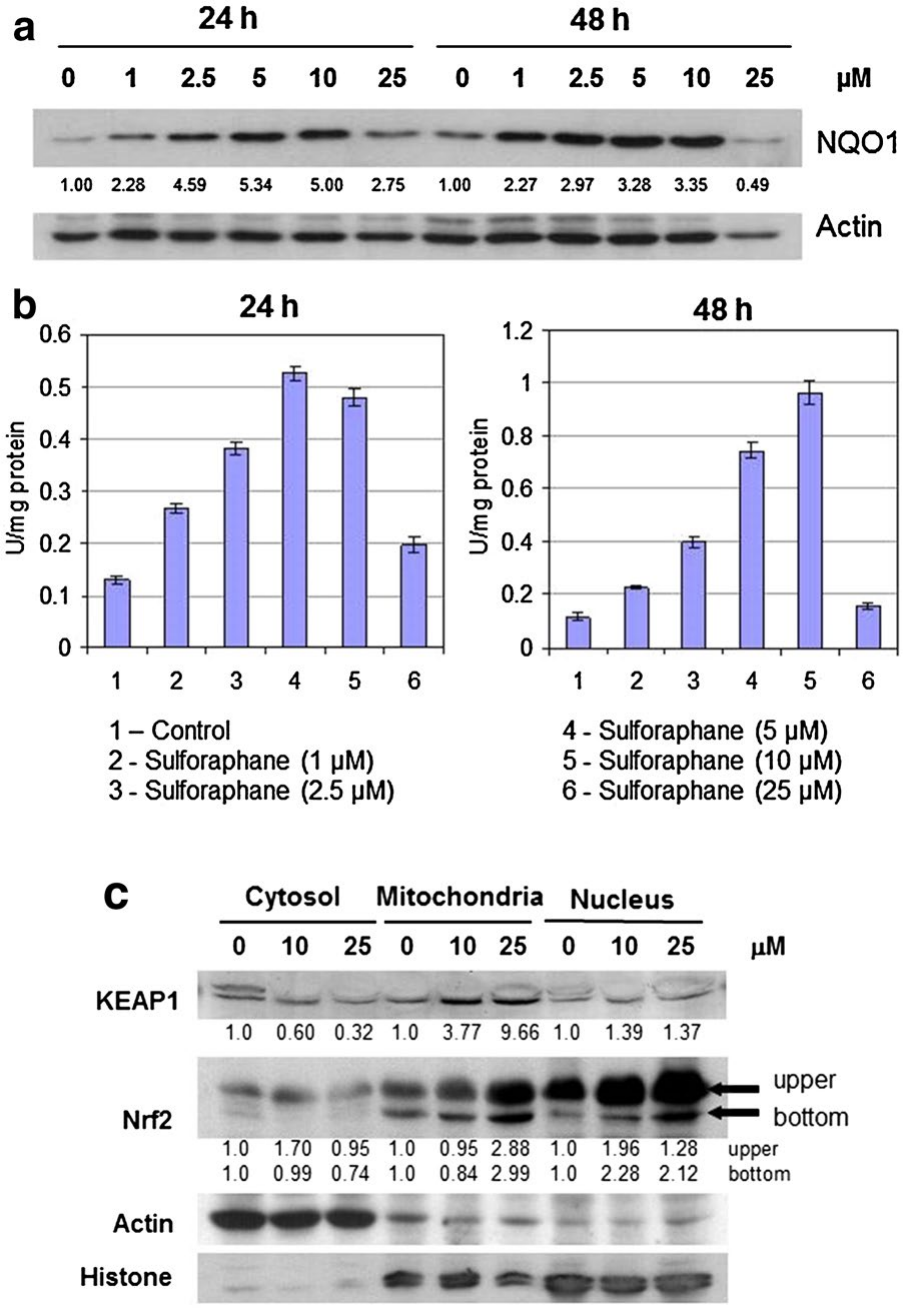
Effect of SFN on NQO1 protein expression and activity. **a** Western blot analysis of NQO1 protein expression in cell lysates treated with 0, 1, 2.5, 5, 10 and 25 μM SFN for 24 and 48 h. **b** NQO1 enzyme activity assay in cell lysates treated with 0, 1, 2.5, 5, 10 and 25 μM SFN for 24 and 48 h. **c** Subcellular distribution of Keap 1 and Nrf2 in the cytosol, mitochondria and nucleus in control and 48 h, SFN-treated HL-60 cells. Actin and histone H1 were used as loading controls for cytosolic and nuclear fractions, respectively. The intensity of specific immunoreactive bands was quantified by densitometry and expressed as fold differences against actin. Values of NQO1 enzyme activities are mean ± SD for three experiments and activities of NQO1 are expressed as µmol of NADPH oxidized**·**min^−1^

**Fig. 2:**
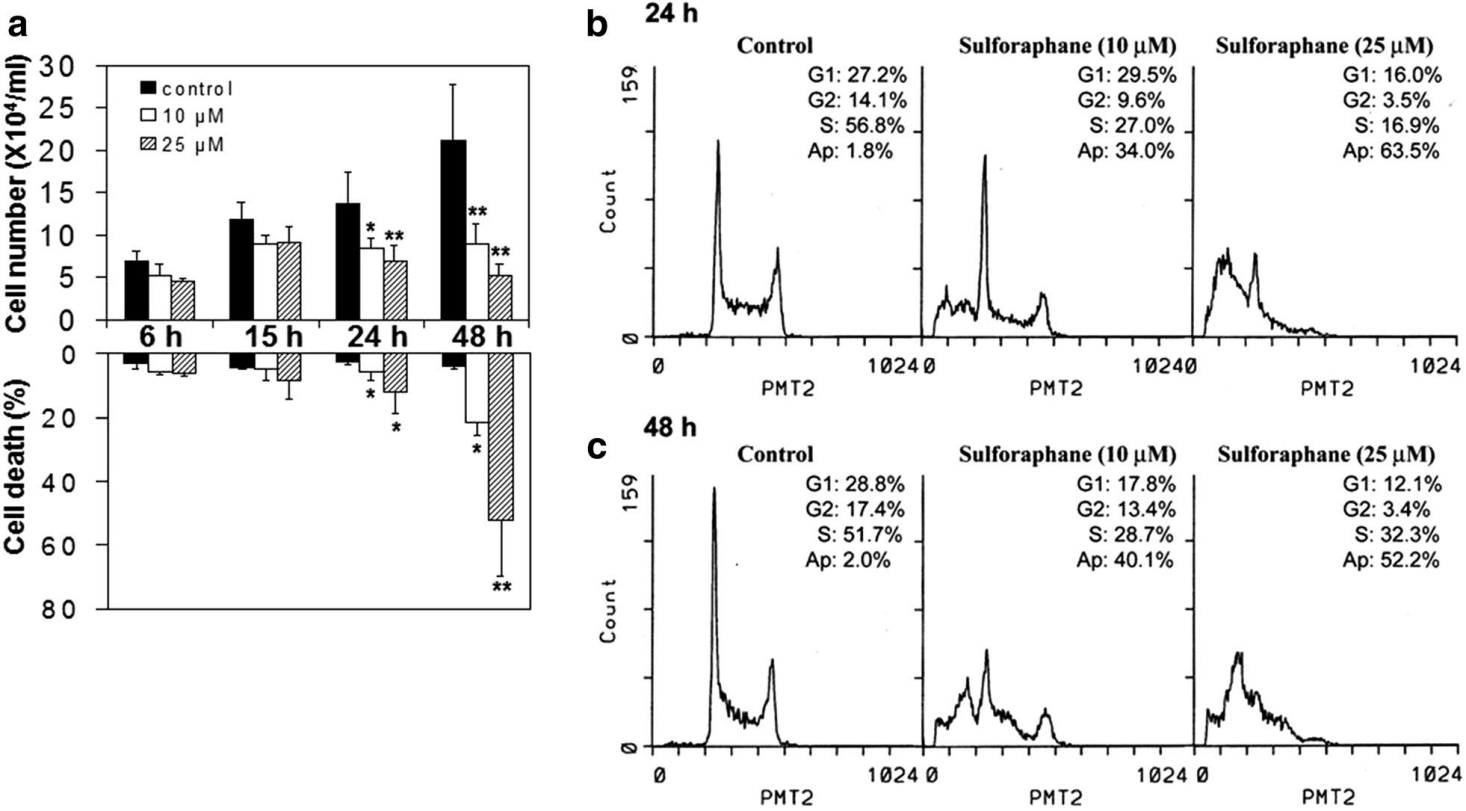
Effect of SFN on cell proliferation, viability and cell cycle control in HL-60 cells. **a** Cells were treated with 0, 10 and 25 μM SFN and the cell numbers were determined at 6, 15, 24 and 48 h by counting using a hemocytometer. Cell viability was measured using the trypan blue exclusion assay. Values are expressed as mean ± SD for three experiments, and *asterisks* * and ** shown above *bars* indicate statistical significance of p < 0.01 and p < 0.001, respectively, compared to vehicle-treated control. **b**, **c** Cells were treated with 0, 10 and 25 μM SFN for 24 and 48 h and analyzed by flow cytometry. The cells in sub-G_1_ phase are used to estimate apoptotic cells

**Fig. 3:**
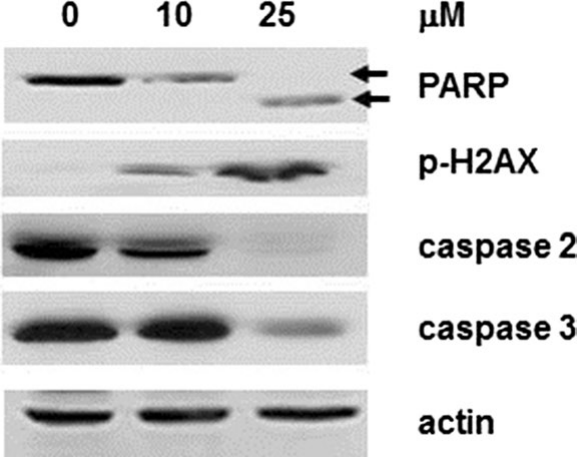
Induction of apoptosis by SFN as determined by changes in PARP cleavage, appearance of p-H2AX, decreases in pro-caspase 2 and pro-caspase 3. Western blot analysis revealed down regulation of PARP expression as evidenced by appearance of the 89-kDa PARP cleavage product, induction of p-H2AX, and down-regulation of both pro-caspase 2 and pro-caspase 3 in ≥ 10 μM, 48 h SFN treated cells

**Fig. 4:**
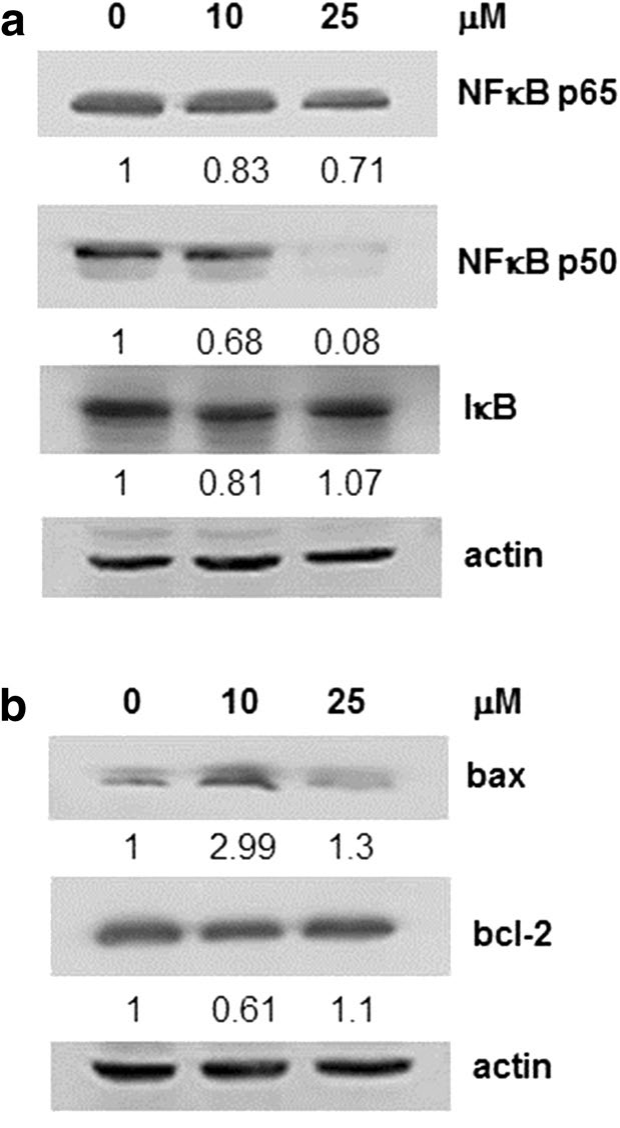
Effect of SFN on the changes of NFκB p65, p50, IκB, bcl-2 and bax expression in HL-60 cells. **a** Western blot analysis revealed the alteration on NFκB subunits p65/p50 and IκB expression in 48 h SFN treated cells. **b** The total protein expression level of bax and bcl-2 was determined by Western blot analysis and the normalized level of cellular bax and bcl-2 and alteration in the ratio of bax to bcl-2 in HL-60 cells treated for 48 h with increasing doses of SFN were calculated. In **a** and **b**, the intensity of the specific immunoreactive bands were quantified by densitometry and expressed as a fold difference against actin (loading control)

Human promyelocytic leukemia cell line (HL-60) was obtained from American Type Culture Collection (Manassas, VA) and maintained in RPMI-1640 supplemented with penicillin, streptomycin and 10 % heat inactivated fetal calf serum, as previously described [36–38]. For treatment, cells were seeded at a density of 1 × 10^5^ cells/ml. SFN dissolved in 1 N NaOH solution and neutralized with HCl, was added to the culture medium to final concentrations of 0, 1, 2.5, 5.0, 10, and 25 μM. Cells were treated with SFN and harvested at different times post-treatment, as indicated in Figs. 1, 2, 3, 4. Cell viability was determined by trypan blue exclusion using a hemocytometer. Cell pellets were stored at −80 °C for biochemical and molecular analyses.

### Preparation of cell extracts for immunoblot analysis

For immunoblotting experiments, cells were lysed in ice-cold RIPA buffer (50 mM Tris, pH 7.4, 150 mM NaCl, 1 mM EDTA, 1 % Triton X-100, 1 % deoxycholate, 0.1 % SDS, 1 mM dithiothreitol and 10 μl/ml protease inhibitor cocktail). Cytosol, mitochondria and nuclear fractions for the analysis of subcellular distribution of Keap1 and Nrf2 were isolated using commercially available kits (Sigma-Aldrich Corporation, St Louis, MO). Protein concentration of cell lysates and fractionated cell extracts were determined by the Coomassie protein assay kit (Pierce Chemical Company, Rockford, IL) using BSA as a standard.

### Immunoblotting

The cell lysates or extracts prepared from cell fractions were resolved by 10 % SDS-PAGE and the proteins were transferred to a nitrocellulose membrane and blocked overnight at 4 °C in TBST buffer (10 mM Tris, pH 7.5, 100 mM NaCl and 0.05 % Tween-20) containing 3 % nonfat dried milk. The blots were incubated with various primary antibodies, followed by incubation for 1 h with appropriate secondary antibodies. The expression levels of actin and histone H1 were used as loading controls. The intensity of the specific immunoreactive bands was detected by enhanced chemiluminescence (ECL), quantified by densitometry and normalized against either actin or histone H1, as previously described [38].

### Cell cycle analysis

HL-60 cells treated with different concentrations of SFN (0, 1, 2.5, 5, 10, and 25 μM) were assayed for changes in cell cycle phase distribution using flow cytometry. Cells were stained with 1.0 μg/ml DAPI (from Sigma-Aldrich Corporation, St. Louis, MO) at pH 6.8, as previously described [38, 39]. DNA content histograms were obtained and the percentage of cells in the respective phases (G1, S and G2/M) of the cell cycle was quantified. Cells undergoing apoptosis were estimated using the appearance of the sub-G1 peak [38, 39].

### Enzyme activity assays

For enzyme activity assays, cells were resuspended in ice-cold 50 mM potassium phosphate buffer (pH 7.4) containing 2 mM EDTA and sonicated. Subsequently, cell suspensions were subjected to centrifugation at 13,000×*g* for 10 min at 4 °C. The resulting supernatant fractions were collected for the measurement of enzyme activities shown below [40].

### NQO1 activity assay

NQO1 activity was measured by the modified method of Lind et al. [41]. Briefly, the reaction was started with the addition of menadione into the reaction mixture containing 33 mM potassium phosphate buffer (pH 7.4), 0.18 mM NADPH, 0.02 % BSA, 0.01 % Tween-20 and cell lysates [40]. The oxidation of NADPH was monitored spectrophotometrically at 340 nm, 25 °C for 2 min with and without 20 μM dicoumarol. The dicoumarol-sensitive portion of the activity was regarded as a measure of NQO1 activity, using the NADPH extinction coefficient (6.22 mM^−1^ cm^−1^) and defined as μmol of NADPH oxidized·min^−1^·mg^−1^ protein.

### Data analysis

The results are expressed as mean ± standard deviation (SD). Differences among groups are assessed by one-way analysis of variance using the SPSS software package for Windows. Data among control and SFN-treated cells are compared using the least significance difference (LSD) test; statistical significance at p < 0.001 or < 0.01 is given respective symbols in the figures.

## Results

### Survey NQO1 status using oncomine

The Oncomine database search was performed to examine the association of NQO1 mRNA expression levels across different sub-type leukemia studies using the reported p values and fold-changes. In two analyses that compared clinical human tissue samples of normal vs leukemia patients, under-expressed NQO1 was found in human acute lymphoblastic leukemia (ALL) and chronic myelogenous leukemia (CML) [42, 43] (Table 1). Since rapid proteolysis of NQO1 occurred in the NQO1*2 isoform [44], low expression of NQO1 found in the individuals with ALL and/or CML most likely carry heterozygous or homozygous NQO1*2 alleles. Several leukemia cell lines are available and can be used as an in vitro cell model to examine the activation of NQO1 expression in NQO1*2 cells. Our studies investigated HL-60 leukemic cells, since previous PCR–RFLP analysis for the SNP (C609T) demonstrated that these cells are heterozygous for the NQO1*2 polymorphism [45]. A further search of the oncomine database involving cancer cell line panels indicated that NQO1 expression in HL-60 cells is consistently low or absent (Table 2). Of 6 HL-60 cell studies queried in oncomine—four published [46–49] and two unpublished (Compendia and Wooster studies), low NQO1 expression consistently ranked in the top 1–4 % of under-expressed genes [47]; this significant reduction in NQO1 can be attributable to the presence of genetic polymorphisms on C609T mutation [22, 44, 45, 50, 51]. Thus, our interrogation of the Oncomine microarray dataset platform provides support that HL-60 cells can serve as an appropriate experimental model to study the activation of NQO1 expression and function by broccoli-derived SFN.

**Table 1:** Comparison of NQO1 expression across 2 clinical specimens as compared to levels in healthy tissue

Under-expressed NQO1				
Cancer type	Gene rank (%)	p value	Fold change	Reference
ALL	Top 1	8.27E−5	−1.138	Kirschner-Schwabe [45]
CML	Top 5	1.29E−10	−1.619	Radich [46]

The mRNA expression levels of NQO1 were compared in normal vs different clinical sub-types of leukemia patients using the reported gene ranks, p values, and fold changes. The clinical specimen fold-change is defined as change in NQO1 mRNA expression level in the leukemia tissue compared to NQO1 mRNA expression level in corresponding normal tissue. Gene rank orders are generated based on the p values obtained in differential gene expression analysis. The gene expression data retrieved from the Oncomine compendium is log transformed and the standard deviation was normalized to one

**Table 2:** Comparison of NQO1 expression in HL-60 across 6 analyses using human cancer cell lines

Under-expressed NQO1				
Cell line	Gene rank (%)	p value	Fold change	Reference
HL-60	Top 1	1.76E−98	−3.978	Garnett [48]
HL-60	Top 2	1.08E−4	−7.291	Compendia (not published)
HL-60	Top 2	1.09E−4	−7.395	Shankavaram [49]
HL-60	Top 2	1.73E−62	−11.959	Barretina [47]
HL-60	Top 2	1.47E−17	−4.235	Wooster (not published)
HL-60	Top 4	8.21E−5	−6.637	Lee [50]

The mRNA expression levels of NQO1 in the HL-60 cell line were investigated. The fold-change is defined as decrease in level of NQO1 mRNA expression in the HL-60 or in different leukemia cells compared to expression levels in non-leukemic cell lines. Gene rank orders are generated based on the p values obtained in differential gene expression analysis. The gene expression data retrieved from the oncomine compendium are log transformed and the standard deviation were normalized to one

### Control of NQO1 in NQO1*2 cells by SFN is accompanied by concomitant change in Nrf2

HL-60 cells were treated with varying doses of SFN for different periods of time. Western blot analysis revealed dose-dependent increases in NQO1 protein expression with the greatest induction occurring after 24 h with 5–10 μM SFN and with 1 μM SFN sufficient to more than double NQO1 levels after treatment for 24- and 48-h (Fig. 1a). Whereas, treatment with 25 μM SFN for 48-h had a significant inhibitory effect on NQO1 protein expression. These results are reflective of a hormetic response and suggest that 1–10 μM SFN is able to induce NQO1 levels in HL-60 cells expressing NQO1*2 genotype.

We next examined whether the observed change in NQO1 protein expression correlated with changes in NQO1 enzymatic activity. As shown in Fig. 1b, treatment with 10 μM SFN for 24- and 48-h revealed time-dependent increases in NQO1 enzyme activity. Conversely, SFN treatment at 25 µM for 24- and 48-h resulted in a significant inhibitory effect on NQO1 activity which may correlate with inhibition of NQO1 protein expression that we observed (Fig. 1b). Previous studies show that SFN targets the transcription factor, Nrf2, by de-repressing its Keap1-dependent ubiquitination and enabling stabilization and subsequent nuclear translocation of Nrf2 to induce ARE detoxification enzymes that includes NQO1 [52]. Therefore, we next investigated whether changes in NQO1 activity by SFN are due to alterations in the subcellular distribution of Keap1-Nrf2 in HL-60 cells. Western blot analysis data show that SFN treatment for 48 h down-regulates cytosolic Keap1 accompanied by increases in nuclear Nrf2 in a dose-dependent manner (Fig. 1c). These results suggest that SFN enhances cytosol-to-nucleus translocation of Nrf2 which partially supports the induction of NQO1 by SFN via Keap1-Nrf2 changes at 10 μM while the control of NQO1 by SFN at 25 μM remains unclear.

### Control of HL-60 proliferation and cell cycle phase transition by SFN

In addition to SFN controlling expression and activity of NQO1 in NQO1*2 HL-60 cells, we also examined whether SFN affects cell proliferation and viability. Trypan blue exclusion assays were performed and results showed time-dependent growth inhibition of HL-60 cells by SFN after 24- and 48-h with an IC_50_ of approximately 10 μM at 48-h post-treatment (Fig. 2a). In addition, time- and dose-dependent decreases in cell viability were observed following 24- and 48-h treatments with 10 and 25 μM SFN as compared to control cells (Fig. 2a).

Cell cycle analyses were performed to determine whether SFN alters cell cycle distribution. The percent of cells in G_1_, S, and G_2_ phases was analyzed (Fig. 2b). The amount of DNA in sub-G_1_ phase, indicative of DNA fragmentation, was used to estimate cell death. As shown in Fig. 2b, SFN treatment for 24 h caused a substantial decrease in number of cells in S-phase and a concomitant increase in cells accumulating in sub-G_1_ phase (1.8 % in control vs 34 and 63.5 % in cells treated with 10 and 25 μM SFN, respectively). Prolonged (48-h) exposure of HL-60 cells to 10 and 25 μM SFN resulted in a significant decrease of cells in both G_1_- and S-phases, and an accumulation of cells in sub-G_1_ phase (Fig. 2c). These results suggest that SFN inhibits cell proliferation through cell cycle arrest and induces apoptotic cell death.

### Control of apoptotic induction and apoptogenic gene expression by SFN in HL-60 cells

Induction of apoptosis by SFN was revealed by the appearance of sub-G_1_ fraction (Fig. 2); biochemical analysis on SFN-induced apoptosis was further investigated by changes in PARP. Dose-dependent decreases in PARP expression and the appearance of the distinct PARP cleavage product were observed in HL-60 cells treated with 25 μM SFN for 48 h (Fig. 3). Corroborative evidence of induction of apoptosis by SFN was further observed by a dose-dependent induction of p-H2AX, a phosphorylated histone which usually marks double-stranded DNA breaks (Fig. 3a) [53, 54]. In addition, the pro-apoptotic potential of SFN was also examined by changes in caspase activation. Treatment with SFN for 48 h decreases pro-caspase 2 as well as pro-caspase 3 expression, indicative of their conversion to active execution caspases that are critical to the induction of apoptosis (Fig. 3). Taken together, SFN-induced apoptosis in HL-60 cells was supported by induction of p-H2AX expression, PARP cleavage, and caspase mediated cell death.

### Control of anti-apoptotic gene expression by SFN in HL-60 cells

A previous study demonstrated that SFN triggers TNF-á-induced apoptosis through inhibition of NFκB and activation of caspase-3 in leukemia cells [55]. We further examined the control of NFκB by SFN in HL-60 leukemia cells. Cells were treated with 10 and 25 μM of SFN for 48 h and changes on p65/p50 subunits of NFκB and its inhibitor (IκB) were determined. SFN significantly reduced expression of p50 subunits whereas minimum changes in IκB expression were observed (Fig. 4a). Altering the combination of dimers within NFκB can disrupt its transcriptional activity by preventing other transcriptionally active NFκB dimers from binding to κB transcription sites or facilitating recruitment of deacetylases to promoter regions [56]. Thus, SFN inhibits NFκB activity by modifying its heterodimer p65/p50. NFκB regulates the expression of anti-apoptosis proteins including bcl-2, and the ratio between bcl-2 and its corresponding apoptosis agonist, bax regulates a biological pathway responsible for cell death. Accordingly, we further examined the expression of bax and bcl-2 in HL-60 cells in response to SFN treatment [57]. As shown in Fig. 4b, incubation of HL-60 cells with 10 μM SFN for 48 h induced bax over-expression. This was accompanied by a concomitant decrease of bcl-2 which led to an increase in the bax/bcl-2 ratio favoring cell death suggesting that induction of apoptosis by SFN is partially through NFκB mediated down regulation of bcl-2.

## Discussion

SNPs are widely observed in the human population and may vary from nonsynonymous to synonymous to manifestations occurring in the noncoding region of the human genome. SNPs can exert influences on gene promoter activity and hence the transcription of mRNAs as well as their conformation and stability. In addition, SNPs can alter the subcellular localization of mRNAs and/or proteins and hence can manifest clinically as diseases in humans. Silencing of genes due to SNPs is also known to occur in eukaryotes and mammalian systems.

NQO1 is affected by several common SNPs of which the C609T serine-to-proline substitution, known as NQO1*2, shows significant correlation with cancer susceptibility [20, 24]. Genotyped individuals who are homozygous for the NQO1*2 polymorphism have silenced NQO1 expression and almost complete loss of NQO1 enzymatic activity, while those genotyped as heterozygous show reduced levels of NQO1 activity and protein expression. Two human hematopoietic cell lines, HL-60 (promyelocytic leukemia) and Raji (Burkitt’s lymphoma), both show similar, substantial reduction of NQO1 transcript levels [45]; phenotypically, however, a complete loss of NQO1 activity was only evident in Raji, while the HL-60 cells expressed a low level of enzyme activity. One interpretation of these discordant findings is that while the expression of NQO1 is controlled by a transcriptional mechanism in both cell types, in the Raji cell line the dominant control may lie in the post-transcriptional proteolytic degradation of NQO1 and that this regulatory feature is not shared by HL-60 cells [44]. This may be viewed in light of various genetic allele contrast models (C vs T), i.e., dominant (CC vs CT + TT), recessive (CC + CT vs TT), homozygous co-dominant (CC vs TT), and heterozygous co-dominant (CC vs CT). In particular, the NQO1 609CT + TT genotype is more common in ALL patients than in controls. Carriers of the variant T allele exhibit a 2.64-fold increased risk for cancer development [58]. These results are in agreement with previously published studies that report under-expression of NQO1 in leukemia (specifically, HL-60 cells) with low NQO1 transcript levels [59, 60]. Thus, our studies using HL-60 to test and advance the feasibility that isothiocyanates in particular SFN can reactivate *NQO1* gene expression and activity in NQO1*2 cells.

Restoration of NQO1 activity has important implications not only for cytoprotection of cells but also for increasing the sensitivity of cells to chemotherapeutic agents such as β-lapachone or mitomycin C. Traditional metabolic studies on NQO1 have regarded two-electron reductions of quinones to hydroquinones as a detoxification mechanism because it circumvents formation of highly reactive semiquinones. In reality, the chemical nature of the quinone and/or hydroquinone will dictate whether the effect involves a toxification or a detoxification reaction. Two-electron reductions of both synthetic and naturally-occurring quinones can induce toxicity in cancer cells. Thus, NQO1 can mediate a futile two-electron cycling between the oxidized quinone forms of the aforementioned drugs and their two-electron reduced hydroquinone forms to induce tumor cell death [12, 61]. The bioactivation of a quinone-containing prodrug such as mitomycin C by NQO1 can be attenuated or nulled in individuals with the heterozygous (CC vs CT) or homozygous (TT vs CC) NQO1*2 genotype, respectively [62]. Results of the NQO1 enzyme activity assay, in combination with a robust expression of the NQO1 protein (see western blot Fig. 1a, b), clearly show restoration/induction of NQO1 protein expression and activity in cancer types expressing low levels of the protein, specifically in NQO1*2 (HL-60) cancer cells. Thus, the demonstration that low NQO1 expression levels could be substantially increased through the use of chemopreventive agents such as SFN, which has much potential for implication in anticancer prodrug therapy.

In support of prodrug idea and the potential application of NQO1 status as a biomarker for certain cancers, we examined microarray data from the Oncomine database to further investigate the association between NQO1*2 and human cancer. Under-expression of NQO1 transcription has been observed in virtually all lymphoma cancer types. Of note, downregulation of NQO1 was found in follicular lymphoma and Burkitt’s lymphoma [45] and, in a series of three lymphoma cell lines, under-expression of NQO1 transcription was shown to correlate with decreased sensitivity to the chemotherapeutic agents: mitomycin C, fluorouracil, and benzoquinone ansamycins e.g. geldanamycin.

Moreover, several instances of substantially diminished NQO1 levels in human sarcoma samples were also observed. Statistically significant under-expression of NQO1 was discovered in liposarcoma and soft tissue sarcoma. In particular, a difference in NQO1 expression was found between cases of liposarcoma with wild-type or mutant p53. Specifically, the liposarcoma samples with mutant p53 exhibited 3-fold lower levels of NQO1 than those with wild-type p53. These results are comparable with previously published data which show that mutant p53 is associated with diminished levels of NQO1 and other phase II detoxification enzymes [63]. As a result of the potent tumor suppressive functions of wild-type p53, any factor that disrupts p53 stability may attenuate wild-type activity and contribute to cell transformation and carcinogenesis. This is of prime importance because NQO1 stabilizes p53 and prevents its degradation [18, 64–66].

In summary, the present study supports the emerging fields of nutrigenomics and nutrigenetics that describe the ability of specific nutrients to activate intracellular signaling pathways. We demonstrate that low NQO1 expression, as found in human leukemic HL-60 cells harboring the polymorphic NQO1*2 variant can be reactivated by physiologically achievable doses of SFN via a mechanism involving the activation of Nrf2-mediated transcription signaling control. It is noteworthy that in nutrigenomic studies comparing potency of phytochemicals, SFN exhibits the highest potency determined by its “CD value”, defined as the concentration of a compound required to double NQO1 specific activity in Hepa 1c1c7 murine hepatoma cells [67, 68]. The concentration of SFN required to double the activity of NQO1 is as low as 0.2 μM. Although some studies indicate that high doses of phytonutrients provide the greatest cytoprotective effects, our studies confirm the findings of others that lower doses of SFN may have greater efficacy [69].

Diets rich in SFN or its precursor may result in bioavailable amounts that are sufficient to overcome the “CD” threshold required to reactivate NQO1*2 to expression levels comparable to that of the NQO1 genotype for treatment of patients with leukemia. Characterizing responders to neoadjuvant therapy would be particularly beneficial in population groups known to have a high prevalence of the homozygous NQO1*2 genotype [70]. Knowledge of the CD threshold is critical to minimize overtreatment by improving efficacy of cancer chemotherapy and/or avoid unnecessary delay of treatment in alleviating cancer susceptibility. These possibilities warrant further research and are under active consideration.

## Conclusions

Important findings addressed are (1) Oncomine mining is a valuable and useful approach to guide future research, (2) SNP-associated NQO1 levels of expression can serve as a biomarker for certain cancers and (3) SFN can activate NQO1 expression in NQO1*2 cells.

## Abbreviations

NQO1, NAD(P)H: quinone oxidoreductase 1
SFN: sulforaphane
Nrf2: nuclear factor (erythroid-derived 2)-like 2
NFκB: nuclear factor-κB
SNPs: single-nucleotide polymorphisms
Keap1: kelch-like ECH-associated protein 1
PARP: poly(ADP-ribose) polymerase
p-H2AX: phosphorylated histone H2AX
IκB: inhibitor of κB
bcl-2: Bcl-2 (B cell lymphoma 2
BSA: bovine serum albumin
ECL: enhanced chemiluminescence
ALL: acute lymphoblastic leukemia
CML: chronic myelogenous leukemia
ARE: antioxidant response element
CD value: the concentration of a compound required to double NQO1 specific activity in Hepa 1c1c7 murine hepatoma cells
